# The effectiveness of a collaborative infertility counseling (CIC) on pregnancy outcome in women undergoing in vitro fertilization: a randomized trial

**DOI:** 10.1186/s12884-020-03417-6

**Published:** 2020-11-25

**Authors:** Mahboobeh Rasoulzadeh Bidgoli, Robab Latifnejad Roudsari, Ali Montazeri

**Affiliations:** 1 Trauma Nursing Research Center, Kashan University of Medical Sciences, Kashan, Iran; 2 Faculty of Medical Sciences, Tarbiat Modares University, Tehran, Iran; 3 Nursing and Midwifery Care Research Center, Mashhad University of Medical Sciences, Mashhad, Iran; 4 Department of Midwifery, School of Nursing and Midwifery, Mashhad University of Medical Sciences, Mashhad, Iran; 5 Population Health Research Group, Health Metrics Research Center, Iranian Institute for Health Sciences Research, ACECR, Tehran, Iran; 6 Faculty of Humanity Sciences, University of Science and Culture, Tehran, Iran

**Keywords:** Collaborative counseling, Infertility, In vitro fertilization, Pregnancy

## Abstract

**Background:**

The optimal objective of infertility treatments is to increase pregnancy rate. The aim of this study was to assess the effectiveness of a collaborative counseling program on pregnancy rate in women undergoing in vitro treatment.

**Methods:**

This was a parallel group randomized trial on a sample of 60 women attending to an infertility research center affiliated to Mashhad University of Medical Sciences for fertility treatment. Women were randomly assigned to an intervention or a control group. Then, a five-session program offered to the intervention group while the control group received nothing expect the usual care. The primary outcome for the study was positive pregnancy test at the end of study. Statistical analyses including independent samples t-test were performed to explore the data.

**Results:**

The outcome analysis showed that there were no significant differences in pregnancy rate between the intervention and the control groups (*P* = 0.298). Also, there were no significant differences in follicle and embryo numbers between two groups. However, a significant difference was observed between two groups in terms of oocyte numbers where the intervention group had more oocyte (*P* = 0.014).

**Conclusion:**

Overall the findings indicated that the collaborative infertility counseling did not improve treatment success in infertile women undergoing in vitro fertilization.

**Trial registration:**

IRCT201110267915N1. Registered 2014.07.25-Retrospectively registered (http://en.irct.ir/trial/8359).

## Background

Infertility is a global problem that affects many people worldwide [1–3]. It is believed that several factors might cause infertility including psychological factors [4–14]. As such studies have shown that anxiety, depression and stress can affect the number of oocyte, embryos and positive pregnancy test leading to changing the hormone levels that are associated with human fertility [12, 15–18]. Thus current practice on infertility treatment is focusing on integrative approaches that include infertility counseling [19, 20]. This is a novel theory that synthesizes medical and psychological aspects of reproductive health [1].

As such Covington purposed a framework, namely the collaborative reproductive healthcare model [1]. This model considers all physiological, psychological, and social issues that might be relevant to infertile individuals. In the collaborative reproductive healthcare model, midwives, psychiatrists, physicians and gynecologists work together and take care for infertile patients right from the start to the final stage of treatment [1]. Investigators that pursuing this collaborative counseling model reported that it could reduce infertility-related stress and enhance the marital satisfaction among infertile women. They suggested that this model could be a useful intervention to mange stress and enhance coping strategies in this population [21–23]. However, a few studies exist that evaluated the effectiveness of psychosocial interventions in terms of infertility outcomes [9, 24–28]. Thus this study aimed to investigate the effectiveness of the collaborative infertility counseling (CIC) on pregnancy rate in women undergoing in vitro fertilization (IVF).

## Methods

### Design and the study sample

This was a parallel group randomized trial in order to assess the effectiveness of a collaborative infertility counseling (CIC) on pregnancy outcome in infertile women attending a referral hospital affiliated to Mashhad University of Medical Sciences in Mashhad, Iran. Infertile women were included in the study if they met the following criteria: being Iranian, ability to read and write, absence of any physical and mental disorders (as indicated by asking women to specify if they receive any medications), not being smoker, not receiving oocyte donation and not being a gestational surrogate. The exclusion criteria were: having ovarian hyper stimulation syndrome, lack of ovarian response to the fertility medications (at least for 3 mature follicles with size of 18–20 mm) and leaving the treatment for any reason.

### Intervention

The intervention consisted of five sessions of individual counseling including topics on the causes and treatments of infertility, communication with family and health care providers, coping skills, and stress management. A midwife (the first author, two sessions), a gynecologist (one session) and a clinical psychologist (two sessions) offered the counseling program. Each session lasted for about 1 h, and was held during IVF treatment cycles. At first meeting an audio CD, a leaflet on relaxation techniques, and a checklist to record relaxation practice were given to each participant. The detailed structure of the sessions is reported elsewhere [22, 23]. The control group only received the IVF treatment similar to the intervention group. However, after the completion of the study all patients in the control group received the educational pamphlet and the audio CD.

### IVF treatment

All patients in two groups received a similar two consecutive IVF treatment cycles, which offered with a 28-days interval. The IVF treatment consisted of ovarian stimulation, oocyte retrieval, sperm retrieval, fertilization, embryo development, embryo transfer and final blood pregnancy test. As such the intervention was implemented between two treatment cycles.

### Outcomes

The primary outcome was positive pregnancy test as indicated by the hcG blood pregnancy test. Laboratory personnel who were not connected to the study carried out all tests 2 weeks after completion of intervention in the same hospital. The secondary outcome was the number of follicles, oocytes and embryos. A sonographist and a laboratory technician determined follicle, oocyte and embryo numbers, respectively.

### Sample size

The required sample size for the study was calculated based on expected outcomes and our own systematic review of the literature [29]. The following formula was used to calculate the sample size [30]:

$$ \mathrm{n}={\left({\mathrm{Z}}_{\alpha /2}+{\mathrm{Z}}_{\upbeta}\right)}^2\ast \mathrm{P}\;\left(1-\mathrm{P}\right)\ast \left(\mathrm{r}+1\right)/{\left(\mathrm{P}1-\mathrm{P}2\right)}^2 $$

Where the following parameters were considered: Z_α/2_ = 1.96 and Z_β_ = 0.842 (for 5% significance level and power 80%); P1 = 0.05 (expected outcome in control group), P2 = 0.30 (expected outcome in intervention group); *r =* 1 (equal cases per each group), P = P1 + rP2/r + 1 = 0.175. As such we estimated that the study would require at least 36 infertile women per each group. Considering 10% drop out a sample of 40 women per each group was thought.

### Randomization

The first participant was allocated to the control group through coin tossing, and then, the second women assigned to the intervention group. This was continued for every participant until the required sample size for each group was achieved. Since the main investigator was responsible for providing counseling thus there was no possibility for blinding.

### Statistical analysis

We used intention-to treat analysis and thus the data analyses were performed for the study groups based on the initial random allocation. The Kolmogorov-Smirnov test was used to assess the normality of data distribution. Descriptive statistics including mean, standard deviation, frequencies and percentages were used to explore the data. Independent sample t-test, Chi-Square or Fisher’s exact test and Mann-Whitney U test (where necessary) were used for group comparison. *P*-value ≤0.05 was considered statistically significant. The SPSS software version 11.5 (SPSS, Inc. Chicago, Illinois, USA) was used to perform statistical analyses.

## Results

**Fig. 1:**
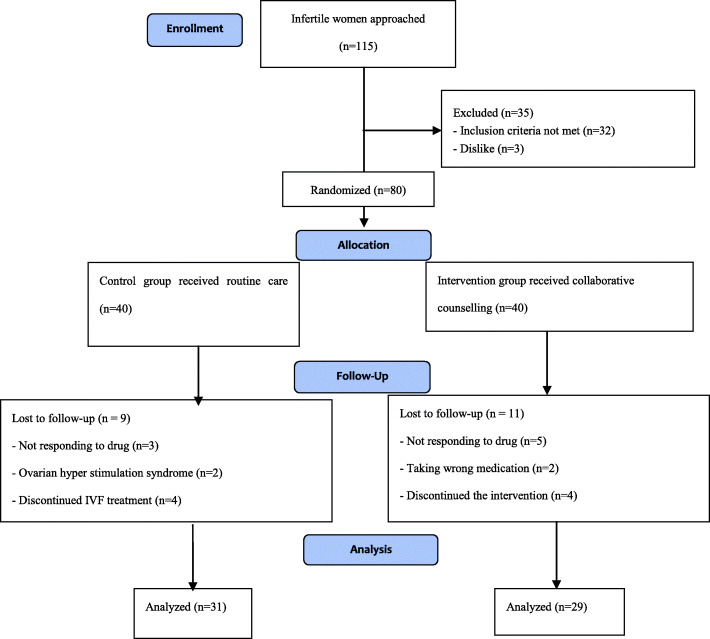
The study flowchart

In all 115 women were approached. Of these, eighty women met the inclusion criteria and randomly were assigned into the study groups (intervention and control). However, the final sample consisted of 60 women including 29 women in the intervention group and 31 women in the control group (Fig. 1).

The two groups were not significantly differed in terms of demographic characteristics, and infertility-related data (Table 1).

**Table 1:** Characteristics of the participants in two groups

	Control (***n*** = 31)	Intervention (***n*** = 29)	P*
	No. (%)	No. (%)	
**Age groups**			0.169
20–24	4 (12.9)	3 (10.3)	
25–29	17 (54.8)	11 (37.9)	
30–34	8 (25.8)	7 (24.1)	
35–40	2 (6.5)	8 (27.6)	
**Education**			
Primary	8 (25.8)	4 (13.8)	0.109
Secondary	17 (54.9)	16 (55.1)	
Higher	6 (19.4)	9 (31.0)	
**Cause of infertility**			0.597
Male	16 (51.6)	10 (34.5)	
Female	8 (25.8)	9 (31.0)	
Both	3 (9.7)	4 (13.8)	
Unknown	4 (12.9)	6 (20.7)	
	**Mean ± SD**	**Mean ± SD**	
**Duration of infertility (year)**	6.08 ± 4.31	5.62 ± 4.26	0.629
**Duration of treatment (year)**	4.03 ± 4.24	3.83 ± 3.97	0.934
**Number of IUI cycles**	1.16 ± 1.43	1.34 ± 1.34	0.480
**Number of IVF cycles**	0.32 ± 0.54	0.44 ± 1.18	0.530

*Derived from chi-square and Mann-Whitney U test as necessary

*IUI* intra uterine insemination, *IVF* in vitro fertilization

As demonstrated in Tables 2, 31% of participants in the intervention group and 45.2% of participants in the control group had positive pregnancy test. However, there was no significant difference between the two groups with regard to positive pregnancy test (*P* = 0.298).

**Table 2:** Differences in the pregnancy test between two groups

	Control (***n*** = 31)	Intervention (***n*** = 29)	P*
	No. (%)	No. (%)	
Positive	14 (45.2)	9 (31)	
Negative	17 (54.8)	20 (69)	
			0.298

*Derived from Fisher’s exact test

The independent samples t-test showed that the two groups were not statistically differed with regard to the number of follicle and embryo but the number of oocytes was statistically different between the two groups at the end of the study (*P* = 0.014) (Table 3).

**Table 3:** Comparison of treatment outcomes in two groups

	Control (*n* = 31)	Intervention (*n* = 29	P*
	Mean ± SD	Mean ± SD	
Follicle number	13.50 ± 4.4	15.1 ± 5.3	0.242
Oocyte number	8.0 ± 3.8	10.9 ± 4.3	0.014
Embryo number	5.8 ± 2.9	7.3 ± 3.2	0.082

*Derived from independent samples t-test

## Discussion

Overall the findings of this randomized trial showed that collaborative infertility counseling did not improve pregnancy rate, although the number of follicles, oocytes and embryos in the intervention group was increased.

As suggested the collaborative reproductive healthcare model is a patient-centered model of care in infertile women [31]. In this regard the success or failure of this model might be related to other approaches where psychosocial interventions were used to improve pregnancy rates in this population. For instance, in a systematic review, Boivin et al. indicated that pregnancy rates were unlikely to be affected by psychosocial interventions. They suggested that more investigations are needed to prove that psychosocial interventions could improve pregnancy rate. However, the same systematic review acknowledged that three of eight studies showed a significant increase in pregnancy rate within 3 to 18 months following a number of psychosocial interventions [32]. On the contrary, Hamerli et al. in a meta-analysis reported that despite the absence of clinical effects on mental health measures, psychological interventions might increase the pregnancy likelihood among infertile women [33]. One explanation for such observation might relate to the fact that elevation in pregnancy rate was due to improvement in couples’ sexual relationship in intervention group.

There is evidence that relaxation can lead to increased pregnancy rate after IVF treatment [17]. Perhaps relaxation could reduce stress and increase pregnancy rate. As such Ramezanzadeh et al. reported that a psychiatric intervention including 6 to 8 sessions of psychotherapy improved pregnancy rates in infertile couples [12]. In another study Li et al. reported that pregnancy rates increased after 6 months follow up in their research [11]. In Li study, participants reported best quality of sleep. Perhaps this was helpful for promoting infertile women’s self-compassion, adaptive emotion regulation and infertility-related coping strategies, which, in turn, influenced the pregnancy rates [11]. However, Kapfhamer et al. reported that patients’ stress related to infertility most likely does not affect a possible pregnancy [34]. Similarly, a meta-analysis on emotional distress in infertile women and failure of assisted reproductive technologies concluded that there is no association between pregnancy outcome and pretreatment emotional distressing infertile women [32]. Hashemi et al. reported that even anxiety (low or high level) have no effect on the pregnancy rate after ART treatment [35].

The contradictory results in previous investigations on the effect of stress or anxiety on successful outcomes for infertility treatment were probably consequence of different types of interventions or levels of psychological distress, various methodologies, small and inadequate samples, different instrument applied to assess psychological distress or limitations of studies in adjusting the analysis for potential confounders. However as far as our study concerns the findings suggest that still firm evidence does not exist to support that such interventions could improve pregnancy rate. Indeed, it seems that there is need for multi-center studies with bigger sample size or even a collaborative studies including samples from different countries to provide solid evidence for policy and practice.

This study had some limitations. The main statistical methods used in this study were independent samples t-test, Mann-Whitney U test and Chi-Square, which are too simple for clinical data and small samples. Thus the results should be interpreted with caution. In addition, this work did not evaluate psychological status of women at baseline while could have been evaluated the psychological status such as stress and anxiety at baseline to see if women were similar in these respects. As such it is recommended that the future studies perform such assessments at baseline. Finally, one should note that we used limited pregnancy outcomes while it is suggested that the miscarriage rate and continuation of pregnancy rates also should be considered, as they are very important outcomes.

## Conclusion

Overall the findings indicated that the collaborative counseling did not improve pregnancy rate in infertile women. However, it might increase oocyte numbers that in turn could be a sign for an increased chance of pregnancy.

## Abbreviations

CIC: Collaborative infertility counseling
IVF: In vitro fertilization
CD: Compact disc
hcG: Human chorionic gonadotropin
